# High-Performance Transmission of Surface Plasmons in Graphene-Covered Nanowire Pairs with Substrate

**DOI:** 10.3390/nano9111594

**Published:** 2019-11-10

**Authors:** Da Teng, Kai Wang, Qiongsha Huan, Yongzhe Zhao, Yanan Tang

**Affiliations:** 1 School of Physics and Electronical Engineering, Zhengzhou Normal University, Zhengzhou 450044, China; 2 Key Laboratory of Infrared Imaging Materials and Detectors, Shanghai Institute of Technical Physics, Chinese Academy of Sciences, Shanghai 200083, China; 3 School of Chemistry and Chemical Engineering, Zhengzhou Normal University, Zhengzhou 450044, China

**Keywords:** graphene plasmons, subwavelength structures, mid-infrared waves, waveguide

## Abstract

Graphene was recently proposed as a promising alternative to support surface plasmons with superior performances in the mid-infrared range. Here, we theoretically show that high-performance and low-loss transmission of graphene plasmons can be achieved by adding a silica substrate to the graphene-covered nanowire pairs. The effect of the substrate layer on mode properties has been intensively investigated by using the finite element method. Furthermore, the results show that inserting a low index material layer between the nanowire and substrate could compensate for the loss accompanied by the substrate, thus the mode properties could be adjusted to fulfill better performance. A reasonable propagation length of 15 μm and an ultra-small normalized mode area about ~10^−4^ could be obtained at 30 THz. The introduction of the substrate layer is crucial for practical fabrication, which provides additional freedom to tune the mode properties. The graphene-covered nanowire pairs with an extra substrate may inspire potential applications in tunable integrated nanophotonic devices.

## 1. Introduction

Surface plasmons (SPs) [1], which are surface electromagnetic waves propagating along a metal-dielectric interface, have been widely investigated for applications of compact and high-performance optical devices [2] far beyond the diffraction limit [3]. Up to now, tremendous progress has been made in plasmon-based photonic integrated circuits (PICs) [4,5,6]. Usually, noble metals are employed to produce plasmonic optical components, such as waveguide [7,8,9,10,11,12,13,14,15,16], laser [17,18], beam splitter [19], coupler [20], switch [21], and modulator [22], to mention a few. However, metal-based plasmonic devices suffer from large Ohmic losses and lack of tunability, thus hindering the applications under some circumstances.

Recently, reports from both theoretical and experimental studies have shown that graphene [23], a two-dimensional carbon material, can also support SPs [24,25]. Unlike metal SPs, graphene plasmons show remarkable optical properties of extremely strong mode confinement, huge field enhancement, and tunability of electromagnetic properties. To some extent, graphene is more suitable for integration in optical devices because of its planar structure. So far, many efforts have been made to develop graphene plasmonic devices, including modulators [26,27], couplers [28,29], switch [30], and waveguides [31,32,33,34,35]. As the key component in PICs, lots of promising graphene-based waveguides have been intensively studied in the past few years [36,37,38,39,40,41,42]. In this kind of device, long propagation length and ultra-small mode size are desired. Particularly, the graphene-covered nanowires (GCNWs) [43,44,45,46,47,48,49,50,51,52,53] have attracted a lot of interest for their simple structure. Experiments [43,44] have shown that the dielectric nanowire can be easily coated by a graphene layer due to van der Waals force. Gao et al. [45] presented an analytical model for plasmon modes in single GCNW. Huang et al. [49] proposed a drop-shaped GCNW for terahertz waveguiding. Li et al. [51] studied the bi-stable scattering in GCNWs. Nevertheless, the plasmon modes in GCNWs are relatively weak confined with normalized mode size about ~10^−3^. To further downscale the mode size, graphene-covered nanowire pairs [54,55,56,57] have been proposed and investigated, and the mode size of which is one order of magnitude smaller compared with that of GCNWs. However, in most previous studies, GCNWs are assumed to be surrounded by air or embedded in low index materials, which means that the GCNWs are suspended without support. For practical PICs applications, a buffer or substrate is essential. Recently, Hajati et al. [47] investigated the influence of buffer on the graphene-coated nanowire, and results showed that the mode properties are greatly improved.

In this work, we theoretically investigate the graphene-covered nanowire pair with an extra substrate, and the influence of the substrate on mode properties of the graphene-covered nanowire pairs is studied. Results show a very good optical performance can be achieved. Also, the dependence of mode properties on the substrate thickness, gap size, nanowire radius, frequency, and chemical potential are studied. It could be expected that this work would be beneficial to the applications of GCNWs in tunable integrated optical devices.

## 2. Theoretical Model and Methodology

**Figure 1:**
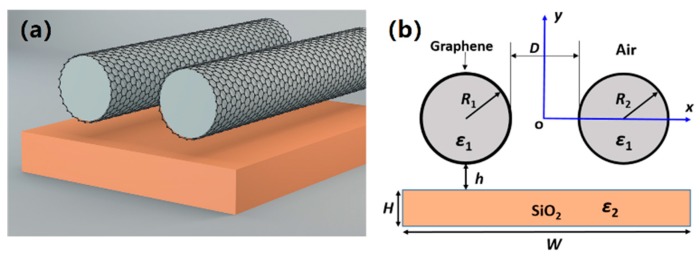
Schematic diagram of the proposed waveguide structure. (**a**) The three-dimensional view; and (**b**) the cross-sectional view.

Figure 1 shows the schematic diagram of the proposed waveguide structure, which is composed of a graphene-covered nanowire pair (with radii of *R*_1_, *R*_2_, and permittivity of *ε*_1_) placed above a SiO_2_ layer (with height and width of *H*, *W*, and permittivity of *ε*_2_ = 2.25) substrate surrounded by air. The thickness of graphene is *Δ* = 0.33 nm. The vertical and horizontal gap distances are *h* and *D* (*D* >> *Δ*), respectively. The permittivity of graphene layer can be obtained by *ε*_g_ = 1 + *iσ*_g_/(*ε*_0_*ωΔ*) [25], where *ω* is the angular frequency and *ε*_0_ is the permittivity in air. The conductivity of graphene, consisting of the interband and intraband contributions, could be derived from the Kubo’s formula [58,59]:

(1) $$\sigma_{g}=\sigma_{\mathrm{intra}}+\sigma_{\mathrm{inter}}$$

(2) $$\sigma_{\mathrm{intra}}=\frac{2ie^{2}k_{B}T}{\pi \hbar^{2}(\omega +i/\tau )}\ln[2\cosh(\frac{\mu_{c}}{2k_{B}T})]$$

(3) $$\sigma_{\mathrm{inter}}=\frac{e^{2}}{4\hbar^{2}}[\frac{1}{2}+\frac{1}{\pi }\arctan(\frac{\hbar \omega -2\mu_{c}}{2k_{B}T})-\frac{i}{2\pi }\ln\frac{{\left(\hbar \omega +2\mu_{c}\right)}^{2}}{{\left(\hbar \omega -2\mu_{c}\right)}^{2}+{\left(2k_{B}T\right)}^{2}}]$$

where *τ* is the electron relaxation time, *T* is the temperature, *μ*_c_ is the chemical potential of the graphene, *ћ* is the reduced plank constant, *k_B_* is the Boltzmann’s constant, and *e* is the charge of the electron. In what follows, we set *τ* = 0.5 ps and *T* = 300 K.

Here, we only consider the first-order mode (the fundamental graphene plasmon mode (FGPM)). Assume that the FGPM propagates harmonically along *z*-direction and the electric field varies as exp(*iβz-iωt*), in which *β* = *k_0_N*_eff_ = 2π*N*_eff_/*λ*_0_ is the complex propagation constant. *n*_eff_ = Re(*N*_eff_) is the effective mode index and *N*_eff_ is calculated by the finite element method (FEM). The transmission length of the FGPM can be defined as *L*_p_ = 1/Im(*β*) and calculated by *L*_p_ = *λ*_0_/[2πIm(*N*_eff_)]. The effective mode size *A*_eff_ is evaluated by *A*_eff_ = ∫∫*W*(r)*d*^2^r/max{*W*(r)} [60], where *W*(r) is the electromagnetic energy density of the FGPM. Here we adopt normalized mode size *A*_eff_/*A*_0_ with *A*_0_ = *λ*_0_^2^/4 to illustrate the mode confinement. Also, figure of merit (FoM) [61] is used to provide a proper assessment for the waveguiding performances, which is defined as Re(*β*)/Im(*β*).

## 3. Results

**Figure 2:**
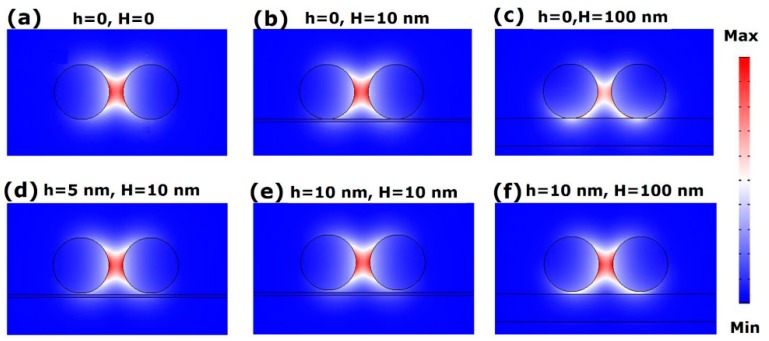
Normalized electric field distributions of the fundamental graphene plasmon mode (FGPM) with different gap sizes and thicknesses of substrate at 30 THz, where *R*_1_ = *R*_2_ = 100 nm, *μ*_c_ = 0.6 eV, *D* = 50 nm, *W* = 1000 nm, *ε*_1_ = 2, and *ε*_2_ = 2.25. (**a**) [*h*,*H*] = [0,0], *N*_eff_ = 22.467 + 0.21479*i*, corresponding to the situation of a graphene-covered nanowire pair in air; (**b**) [*h*,*H*] = [0,10] nm, *N*_eff_ = 22.750 + 0.21675*i*; (**c**) [*h*,*H*] = [0,100] nm, *N*_eff_ = 24.107 + 0.23615*i*; (**d**) [*h*,*H*] = [5,10] nm, *N*_eff_ = 22.685 + 0.21583*i*; (**e**) [*h*,*H*] = [10,10] nm, *N*_eff_ = 22.637 + 0.21529*i*; and (**f**) [*h*,*H*] = [10,100] nm, *N*_eff_ = 23.155 + 0.21576*i*.

**Figure 3:**
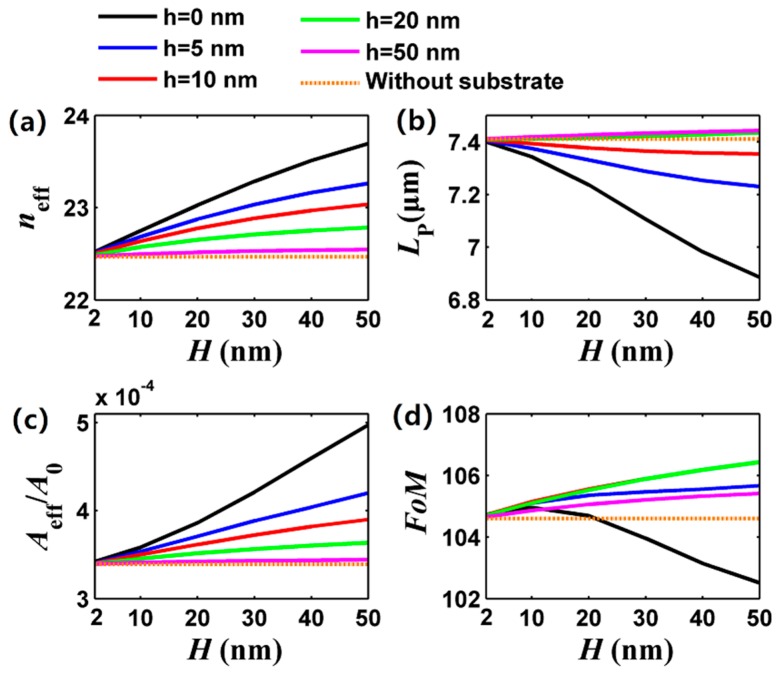
Dependence of the FGPM properties on gap size *h* and substrate thickness *H* at 30 THz. (**a**) Effective mode index; (**b**) propagation length; (**c**) normalized mode size; and (**d**) FoM. Other parameters are the same as Figure 2.

Normalized electric field distributions of the FGPM are shown in Figure 2. When no substrate and gap are used (see Figure 1a), which corresponds to the graphene-covered nanowire pair in air, the strong coupling of SPs at two graphene-dielectric interfaces leads to mode field concentration in the gap of the two nanowires. In Figure 2b, the graphene-covered nanowire pair is directly placed on a silica substrate with *H* = 10 nm. The field profile of the FGPM hardly changes with a silica substrate compared to the case in air. Although under this case, the leaky radiation into the substrate increases the loss of the FGPM, the increment is very small (less than 1%). Then, we further adjust *H* to investigate the influence of the silica substrate on the field distribution. As shown in Figure 2c, when *H* = 100 nm, the modal field changes a lot and propagation loss is further enhanced (about 9%). The field distributions of the FGPM with the same substrate thickness of *H* = 10 nm but different vertical gap *h* are shown in Figure 2d,e. Compared with the case of Figure 2b, the mode propagation loss is reduced (smaller Im(*N*_eff_)). This indicates that although the loss is enhanced by adding a substrate, the loss could be compensated by inserting a low index material (In this case, we set *ε* = 1) in the vertical gap. The result is consistent with the report from [47]. As shown in Figure 2f, when further increasing the thickness of the silica substrate to 100 nm (e.g., [*h*,*H*] = [10,100] nm), the mode propagation loss is increased by 0.2% compared to Figure 2e (e.g., [*h*,*H*] = [10,10] nm). The results illustrate that as long as a low index material is inserted in the vertical gap, the thickness of silica substrate has very little effect on mode propagation length. For instance, in Figure 3b, when we set *h* = 5, 10, 20, 50 nm and *H* ranges from 20 nm to 50 nm, the propagation lengths are all about 7.3 μm.

Figure 3 shows the effective mode index, propagation length, normalized mode size, and FoM of the FGPM as a function of the substrate thickness *H* for different vertical gap sizes. Here, the substrate thickness varies from 20 to 50 nm, and a graphene-covered nanowire pair in air (*H* = 0) is also involved for comparison (dotted brown curves). For the vertical gap size *h* = 0 (solid black curves), the nanowire pair is located directly on the substrate, which leads to strong coupling. Increasing *H* results in both larger effective mode index and loss as well as worse waveguiding performance (smaller FoM).

However, the situation is quite different when inserting a low index material (Here, *ε* = 1) between the nanowires and the substrate. As we stated earlier, the low index material layer could compensate for the loss introduced by the silica substrate. For instance, *L*_p_ is 7.1 μm for [*h,H*] = [0,30] nm, while *L*_p_ is 7.4 μm for [*h,H*] = [20,30] nm (see Figure 3b). As seen from Figure 3c, the mode size increases by less than 1-fold for increasing *H*, and trends to that of a free-standing graphene-covered nanowire pair in the air when enhancing *h.* As obtained from Figure 3d, the FoM is around 105 for *h* ≠ 0. For compact integration, smaller vertical gap size *h* and substrate thickness *H* (10 to 20 nm) are preferred.

**Figure 4:**
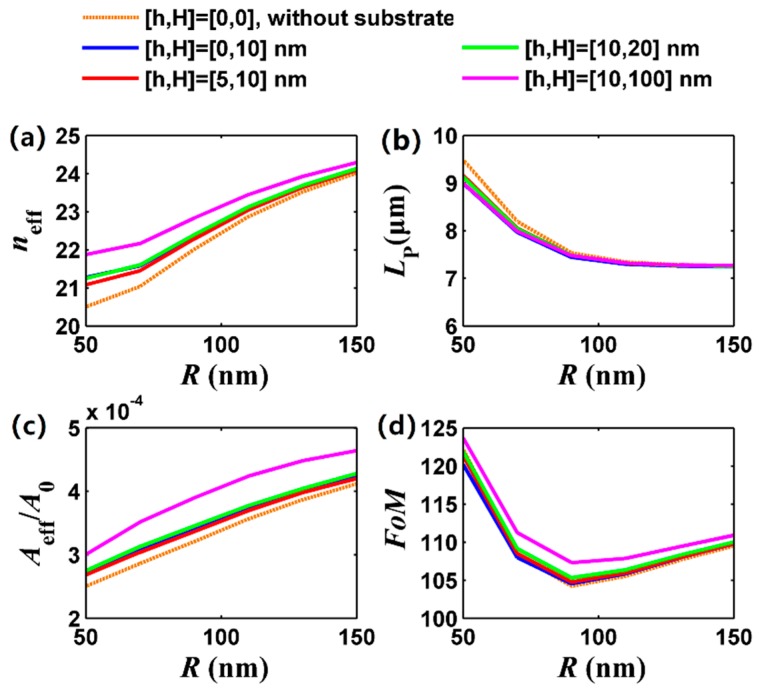
Dependence of the FGPM properties on nanowire radius *R* (*R*_1_ = *R*_2_ = *R*) at 30 THz. (**a**) Effective mode index; (**b**) propagation length; (**c**) normalized mode size; and (**d**) FoM. The dotted brown curve for [*h*,*H*] = [0,0], solid blue curve for [*h*,*H*] = [0,10] nm, solid red curve for [*h*,*H*] = [5,10] nm, solid green curve for [*h*,*H*] = [10,20] nm, and solid pink curve for [*h*,*H*] = [10,100] nm. Other parameters are the same as Figure 2.

Figure 4 shows the FGPM properties with respect to nanowire radius. It can be seen from Figure 4a that the effective mode index increases with increasing nanowire radius, and *n*_eff_ is larger for a thicker substrate under the same nanowire radius, which is consistent with that of Figure 3a. For a smaller radius (50 to 70 nm), the FGPM has a longer propagation length and larger FoM (see Figure 4b,d), which could be verified by previous work [55]. At the same time, we can see that the solid blue, red, green, and pink curves are almost overlapped in Figure 4b, which in turn indicates that the low index material layer could compensate for the loss introduced by the silica substrate. From Figure 4c, we understand the increase of nanowire radius leads to a larger mode size. By comprehensive consideration, a smaller radius could offer better waveguiding performance of the FGPM.

**Figure 5:**
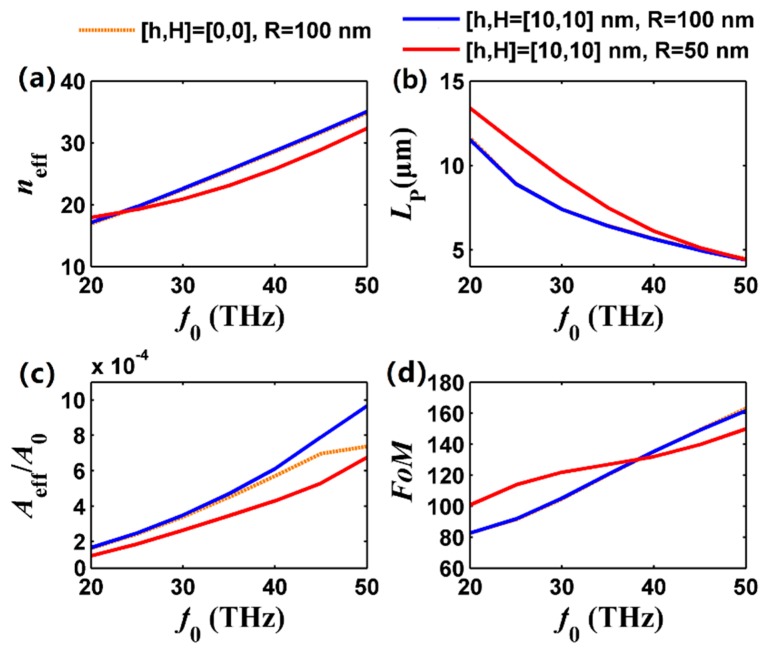
Dependence of the FGPM properties on frequency. (**a**) Effective mode index; (**b**) propagation length; (**c**) normalized mode size; and (**d**) FoM. The dotted brown curve for [*h*,*H*,*R*] = [0,0,100] nm, solid blue curve for [*h*,*H*,*R*] = [10,10,100] nm, and solid red curve for [*h*,*H*,*R*] = [10,10,50] nm. The frequency ranges from 20 THz to 50 THz. Other parameters are the same as Figure 2.

Figure 5a demonstrates the dispersion relations of the FGPM in the graphene-covered nanowire pair, and the effective mode index increases monotonically with frequency increasing. At higher frequencies, the interband contribution of *σ*_g_ is high [24,47], leading to a higher propagation loss (see Figure 5b). In fact, suffering from high absorption of the graphene layer in the mid-infrared band, the propagation length of this kind of waveguide is limited to about 10 to 20 μm based on different *L*_p_ definitions. Under the same circumstance, a graphene-covered nanowire pair with smaller radius shows longer *L*_p_ (see Figure 5b) and smaller mode size (see Figure 5c), which is consistent with Figure 4. The mode size is increased about 6-fold when *f*_0_ ranges from 20 to 50 THz, and the introduction of a substrate and air gap slightly affect the mode size (see dotted brown and solid blue curves in Figure 5c). Noticeably, we find that the dotted brown curve and solid blue curve are nearly overlapped in Figure 5 except higher frequencies in Figure 5c. This means that although a substrate is added to the graphene-covered nanowire pair, the excellent optical performance could be maintained, which illustrates the feasibility of practical applications of the graphene-covered nanowire pair with a substrate.

**Figure 6:**
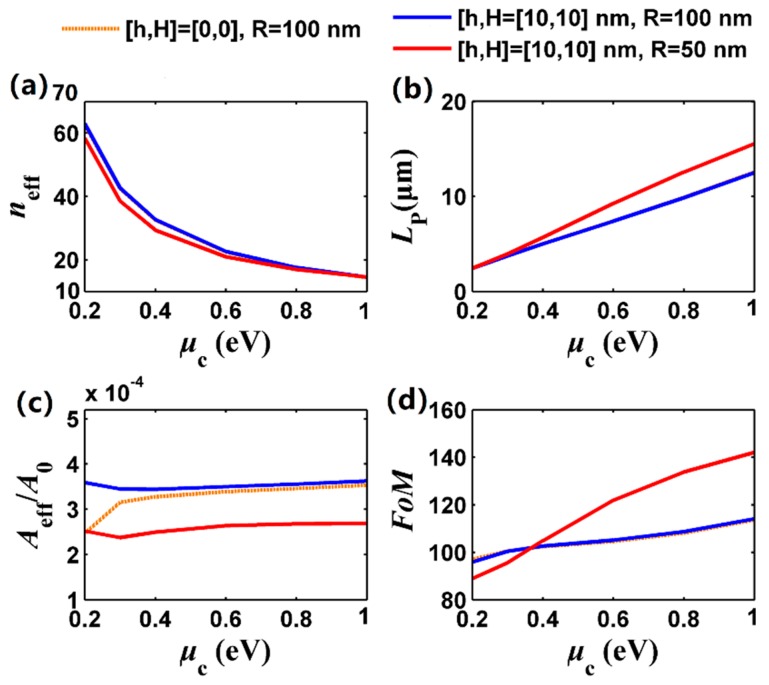
Dependence of the FGPM properties on chemical potential. (**a**) Effective mode index; (**b**) propagation length; (**c**) normalized mode size; and (**d**) FoM. The dotted brown curve for [*h*,*H,R*] = [0,0,100] nm, solid blue curve for [*h*,*H,R*] = [10,10,100] nm, and solid red curve for [*h*,*H,R*] = [10,10,50] nm. *f*_0_ is 30 THz and *μ*_c_ ranges from 0.2 eV to 1 eV. Other parameters are the same as Figure 2.

The tunability of the waveguiding performance is of great importance for practical applications. As the FGPM is very sensitive to the surface conductivity of graphene, it offers us a feasible approach to control the modal behaviors by changing the chemical potential. Figure 6 shows the dependences of the FGPM on the chemical potential at *f*_0_ = 30 THz. When the chemical potential increases from 0.2 to 1 eV, the effective mode index rapidly decreases (see Figure 6a), while *L*_P_ almost linearly increases (see Figure 6b). Again, the graphene-covered nanowire pair with a smaller radius shows better performance. The normalized mode area is almost invariable when *μ*_c_ ranging from 0.2 to 1 eV. For *μ*_c_ = 1 eV, *L*_P_ and *A*_eff/_*A*_0_ are 12.5 μm and 3.6 × 10^−4^ at *R* = 100 nm, respectively. Finally, the increase of *μ*_c_ leads to an increase of FoM. Also, we find that the dotted brown curve and solid blue curve are nearly overlapped, except smaller chemical potential in Figure 6c. This indicates the graphene-covered nanowire pair with a thin substrate layer could have similar optical performance compared with the suspended graphene-covered nanowire pair in air.

## 4. Discussion

As we have shown before, adding a dielectric substrate to the graphene-covered nanowire pairs is crucial for practical fabrication, and provides additional freedom to fulfill better waveguiding performance. Finally, we investigated the influence of different substrate materials on the waveguiding properties, and the results can be seen in Table 1. Here, *R*_1_ = *R*_2_ = 50 nm, *D* = 50 nm, [*h*,*H*] = [0,10] nm, *μ*_c_ = 1 eV, *T* = 300 K, *τ* = 0.5 ps, *ε*_1_ = 2, and *f*_0_ = 30 THz. When the substrate permittivity ranges from 2.25 to 12.25, the propagation loss increases and *L*_p_ decreases. However, the stronger mode field confinement (smaller mode area) can be obtained for larger permittivity. Therefore, to achieve long range propagation, smaller permittivity is preferred. 

At last, we need to state that here we omit the study of the influence of the nanowire permittivity and horizontal gap distance *D* on mode properties, and we just set nanowire permittivity to be *ε*_1_ = 2, which is based on previous work that smaller nanowire permittivity results in better waveguiding properties of a graphene-covered nanowire [45,55]. Also in our previous work [55], we have obtained that increasing *D* could lead to an increase in the mode size as well as the decoupling of the FGPM. Therefore, a moderate horizontal gap distance 50 nm is considered here. In the meantime, the vertical gap size *h* should not be very large, since a larger gap size will lead the decoupling of the hybrid plasmonic mode [60].

## 5. Conclusions

To conclude, we have shown that a very good waveguiding performance could be obtained by using the graphene-covered nanowire pairs with an extra substrate. The dependence of mode properties on the substrate thickness, gap size, nanowire radius, frequency, and chemical potential are intensively studied. By careful design, an FGPM with a very small mode size and large propagation distance could be obtained in the Mid-IR. Particularly, results show that adding a low index material layer between the nanowire and the substrate would compensate for the loss introduced by the substrate. The manipulation of graphene plasmons far beyond the diffraction limit may have potential applications in nanophotonics and photonic integration circuits.

## Figures and Tables

**Table 1:** Comparison of waveguiding performance with different substrate materials.

Substrate Permittivity *ε*_2_	*L*_p_/μm	*A*_eff_/*A*_0_	FoM
2.25	15.23	2.75 × 10^−4^	140.9
4	14.41	2.42 × 10^−4^	138.3
6	13.46	9.61 × 10^−5^	134.4
8	12.48	4.53 × 10^−5^	129.6
12.25	10.28	1.19 × 10^−5^	116.7
